# Topical Steroid-Induced Cushing Syndrome in an 18-Month-Old Child: A Case Report

**DOI:** 10.7759/cureus.109303

**Published:** 2026-05-20

**Authors:** Salman Almansour

**Affiliations:** 1 Pediatric Endocrinology, Qassim University, Buraydah, SAU

**Keywords:** cushing syndrome, diaper dermatitis, hpa axis suppression, infantile obesity, topical corticosteroids

## Abstract

Topical corticosteroids are widely used in pediatric dermatology and are generally considered safe when used appropriately; however, prolonged or inappropriate use, particularly of high-potency agents, may lead to significant systemic adverse effects. This case report describes an 18-month-old female who presented with rapid weight gain and cushingoid features following prolonged application of clobetasol propionate for diaper dermatitis. Clinical evaluation revealed generalized obesity, moon facies, facial plethora, and dorsal hypertrichosis, while laboratory investigations demonstrated markedly suppressed afternoon serum cortisol levels (~2 µg/dL; pediatric reference range: 3-13 µg/dL) and plasma adrenocorticotropic hormone (ACTH) levels <5 pg/mL (pediatric reference range: 10-60 pg/mL), consistent with hypothalamic-pituitary-adrenal axis suppression. Imaging studies exclude adrenal pathology. Discontinuation of the offending agent and close clinical monitoring resulted in gradual improvement of clinical features and partial recovery of HPA axis function. This case underscores the potential for potent topical corticosteroids to induce systemic complications in infants and highlights the importance of cautious prescribing, caregiver education, and early recognition of steroid-related adverse effects in pediatric patients.

## Introduction

Topical corticosteroids constitute a cornerstone of dermatologic therapy for a broad spectrum of cutaneous disorders, particularly inflammatory conditions [1]. Topical corticosteroids exert their pharmacologic effects predominantly through interaction with intracellular glucocorticoid receptors, subsequently binding to response elements within host DNA, reducing pro-inflammatory chemical synthesis, and mitigating inflammation [2-3]. Topical glucocorticoids are frequently prescribed for the management of atopic dermatitis in infants and children, and are also utilized in the treatment of other inflammatory dermatoses, including contact dermatitis, pompholyx, and pediatric seborrheic dermatitis [4]. Nonetheless, topical corticosteroids represent a double-edged sword in pediatric dermatology: while they are highly effective in controlling inflammatory dermatoses, their inappropriate or prolonged use may predispose to significant local and systemic adverse effects [5].

Percutaneous absorption of topical corticosteroids is significantly enhanced in infants and young children owing to their thinner epidermal barrier and increased body surface area-to-weight ratio, a phenomenon that is further accentuated in premature neonates with markedly immature skin [6]. Additionally, reduced metabolic clearance of absorbed glucocorticoids in this population potentiates systemic exposure. The combined effect of increased absorption and diminished metabolism predisposes to suppression of the hypothalamic-pituitary-adrenal axis and subsequent reduction in endogenous cortisol production [3,6]. Several factors contribute to enhanced systemic exposure to topical glucocorticoids in the pediatric population, including the use of high-potency formulations, prolonged duration of therapy, application over extensive body surface areas, and inappropriate dosing in the absence of adequate medical supervision [4].

Chronic or inappropriate prolonged application of topical corticosteroids may result in systemic glucocorticoid excess and subsequent development of Cushing syndrome [7]. Cushing syndrome is characterized by a state of pathological hypercortisolism arising from either exogenous glucocorticoid administration or endogenous cortisol excess, leading to a constellation of systemic clinical manifestations [8]. The presentation of symptoms such as rapid or excessive weight gain, characteristic facial changes including *moon facies*, hirsutism, and the development of abdominal striae may indicate underlying hypercortisolism [7]. Topical corticosteroid-induced Cushing syndrome is an uncommon but well-recognized complication in the pediatric population, typically associated with the use of high-potency corticosteroids such as clobetasol and betamethasone, and carries the risk of hypothalamic-pituitary-adrenal axis suppression and potential adrenal crisis upon discontinuation [9].

Early recognition of topical corticosteroid-induced Cushing syndrome in infants is crucial, as timely identification can prevent serious complications such as adrenal suppression and growth disturbances. The risks are particularly heightened with unsupervised or over-the-counter use of potent corticosteroids, underscoring the need for both clinician vigilance and caregiver education regarding appropriate application and dosing. Reporting cases such as this contributes valuable insight to the existing literature, raising awareness of this rare but significant adverse effect and guiding safer prescribing practices in the pediatric population. This documentation of topical corticosteroid-induced Cushing syndrome provides important contributions to safe prescription and the use of topical corticosteroids in the pediatric population.

## Case presentation

An 18-month-old female was referred to the pediatric outpatient clinic for evaluation of rapid and excessive weight gain, with an increase of approximately 6 kg over three months. Her prior growth and developmental milestones had been appropriate, with no notable medical history. The dietary assessment indicated age-appropriate caloric intake, effectively ruling out nutritional causes of obesity. Further history elicited prolonged use of a potent topical class I corticosteroid, clobetasol propionate (Dermovate; GSK (GlaxoSmithKline), London, United Kingdom), initially prescribed for diaper dermatitis four months earlier. Owing to ambiguous guidance regarding treatment duration, the parents applied the cream three to four times daily to the diaper area over the four months. Notably, the onset of rapid weight gain corresponded temporally with this extended steroid exposure, and there was no family history of endocrine disorders.

The general physical examination did not reveal significant findings except generalized obesity. The patient weighed 16 kg, corresponding to more than the 97th percentile for age and gender. Clinical features suggestive of Cushing syndrome were present, including Cushingoid facies with *moon face* and facial plethora, and dorsal hypertrichosis. No striae, bruising, or hyperpigmentation were observed. The temperature, blood pressure, heart rate, respiratory rate, and oxygen saturation were within normal limits. Laboratory assessment revealed markedly suppressed afternoon serum cortisol of approximately 2 µg/dL (pediatric reference range: 3-13 µg/dL) along with plasma adrenocorticotropic hormone (ACTH) levels below 5 pg/mL (pediatric reference range: 10-60 pg/mL), consistent with hypothalamic-pituitary-adrenal axis suppression secondary to exogenous corticosteroid exposure. Although morning cortisol measurement is generally preferred for assessment of adrenal suppression, the markedly reduced afternoon cortisol level in conjunction with suppressed ACTH and characteristic clinical findings strongly supported exogenous corticosteroid-induced HPA axis suppression. Abdominal ultrasonography demonstrated normal adrenal morphology, excluding adrenal neoplasms. In the context of prolonged exposure to a high-potency topical corticosteroid and the characteristic clinical presentation, the patient was diagnosed with iatrogenic Cushing syndrome secondary to topical clobetasol misuse. The corticosteroid was discontinued, and the patient was enrolled in a structured monitoring protocol to mitigate the risk of adrenal crisis during the withdrawal phase. The topical corticosteroid was gradually tapered by reducing the frequency of application from multiple daily applications to once daily for several days, followed by alternate-day application prior to complete discontinuation. Follow-up was conducted primarily through close clinical assessment because repeated laboratory investigations were limited by practical and financial constraints. The patient remained clinically stable throughout the withdrawal period, with no evidence of adrenal crisis or hemodynamic instability. Telephone follow-up performed within one week revealed early improvement in cushingoid facial features, and subsequent follow-up demonstrated gradual regression of the clinical manifestations over time. Subsequent follow-up revealed gradual resolution of cushingoid features and partial restoration of the hypothalamic-pituitary-adrenal axis function.

## Discussion

This case report illustrates the potential for potent topical corticosteroids to induce iatrogenic Cushing syndrome in infants, even when applied for a common condition such as diaper dermatitis. The patient exhibited classical clinical features, including rapid weight gain, Cushingoid facies, and dorsal hypertrichosis, accompanied by biochemical evidence of hypothalamic-pituitary-adrenal axis suppression. The diagnosis was further supported by markedly suppressed serum cortisol and ACTH levels, consistent with exogenous glucocorticoid-mediated suppression of the hypothalamic-pituitary-adrenal axis. The temporal association between prolonged, high-frequency application of clobetasol propionate and the onset of these symptoms underscores the significant systemic absorption that can occur in young children, particularly when potent corticosteroids are applied over occluded areas like the diaper region. This case highlights the importance of recognizing early signs of systemic glucocorticoid excess in the pediatric population to prevent serious complications such as adrenal insufficiency and growth disturbances.

Over several decades, the literature has documented a magnitude of cases of iatrogenic Cushing syndrome secondary to topical corticosteroid use. Most reported cases occurred in pediatric patients, with infancy representing the predominant age group (86%), and diaper dermatitis emerging as the most frequently implicated underlying dermatosis. Percutaneous toxicity from topical corticosteroids correlates directly with the extent of transdermal absorption. Any form of occlusion augments stratum corneum hydration and elevates local temperature, thereby facilitating enhanced drug penetration. Evidence demonstrates that occlusion can amplify systemic absorption by up to tenfold [10].

Published pediatric case reports consistently demonstrate the development of iatrogenic Cushing syndrome in infants exposed to high-potency corticosteroid, clobetasol, over a long period of time for diaper dermatitis [11]. These cases highlight profound hypothalamic-pituitary-adrenal axis suppression and systemic complications secondary to enhanced percutaneous absorption in infancy. Published pediatric case reports have documented severe systemic complications associated with topical corticosteroid-induced iatrogenic Cushing syndrome. In one reported case, an infant succumbed to disseminated cytomegalovirus infection secondary to profound immunosuppression [12], whereas a separate case described cardiovascular complications, including moderate left ventricular hypertrophy and pericardial effusion [13-15].

Cushing syndrome represents an uncommon clinical condition, particularly within the pediatric population, and in children, it most frequently arises secondary to exogenous glucocorticoid administration. Despite documentation in the medical literature, the rarity of Cushing syndrome in the pediatric population leads to failure to promptly recognize the clinical manifestations, delaying diagnosis, and contributing to significant long-term morbidity [16]. Exogenous Cushing syndrome can be diagnosed by 24-hour urinary free cortisol, midnight serum or salivary cortisol levels, and the dexamethasone suppression test, followed by determining ACTH dependence [17].

When cushingoid features develop, clinicians typically implement gradual tapering of glucocorticoid therapy to reduce the risk of relapse of the underlying disease while minimizing the likelihood of glucocorticoid-induced adrenal insufficiency and glucocorticoid withdrawal syndrome. Glucocorticoid tapering should proceed at a rate tailored to maintain remission of the underlying condition; consequently, a universal withdrawal protocol is not feasible. Nevertheless, guidelines for specific pediatric conditions often lack explicit recommendations for assessing recovery of the hypothalamic-pituitary-adrenal axis and may instead suggest evaluating morning cortisol levels only when clinical features of adrenal insufficiency are present [18]. To minimize the risk of developing Cushing syndrome in infants and children using topical glucocorticoids, clinicians should avoid prescribing high-potency corticosteroids for pediatric dermatologic disorders and instead prefer low-potency topical agents when appropriate. Caregivers should receive clear guidance regarding correct application, including limiting use to a thin layer over the affected area and avoiding prolonged or excessive application. Preventive strategies should therefore emphasize cautious prescribing practices, restriction of potent corticosteroids, and thorough parental education regarding the potential adverse effects [19].

## Conclusions

This case highlights the potential for high-potency topical corticosteroids to induce iatrogenic Cushing syndrome in infants and children, emphasizing the necessity for careful prescribing, clear caregiver instructions, and vigilant clinical monitoring. Clinicians must recognize early signs of systemic glucocorticoid excess to prevent serious complications, including adrenal suppression and growth disturbances. The case underscores the risks associated with unsupervised or prolonged use of potent topical agents and reinforces the need for caregiver education regarding appropriate application. Careful follow-up with clinical assessment and, when feasible, serial morning cortisol measurements and ACTH stimulation testing may assist in monitoring recovery of hypothalamic-pituitary-adrenal axis function after withdrawal of potent topical corticosteroids. Future efforts should focus on developing standardized guidelines for pediatric topical corticosteroid use, enhancing awareness of rare but significant systemic effects, and encouraging reporting of similar cases to inform evidence-based dermatologic practice in the pediatric age group.
